# Transcriptomic Profiling of the Tumor Microenvironment in High-Grade Serous Carcinoma: A Pilot Study of Morphologic and Molecular Distinctions Between Classic and SET Patterns

**DOI:** 10.3390/ijms262010229

**Published:** 2025-10-21

**Authors:** Riccardo Giannini, Francesco Bartoli, Katia De Ieso, Tiziano Camacci, Andrea Bertolucci, Lorenzo Piccini, Erion Rreka, Duccio Volterrani, Federica Gemignani, Stefano Landi, Clara Ugolini, Piero Vincenzo Lippolis, Pinuccia Faviana

**Affiliations:** 1 Dipartimento di Patologia Chirurgica, Medica, Molecolare e dell’Area Critica, Università di Pisa, 56126 Pisa, Italy; riccardo.giannini@unipi.it (R.G.); clara.ugolini@unipi.it (C.U.); 2 Dipartimento di Ricerca Traslazionale e delle Nuove Tecnologie in Medicina e Chirurgia, Università di Pisa, 56126 Pisa, Italy; francesco.bartoli@unipi.it (F.B.); duccio.volterrani@unipi.it (D.V.); 3 D.A.I. Oncologia, Azienda Ospedaliero-Universitaria Pisana, 56126 Pisa, Italy; k.deieso@ao-pisa.toscana.it (K.D.I.); t.camacci@ao-pisa.toscana.it (T.C.); 4 D.A.I. Chirurgia Generale e Urologia, U.O. Chirurgia Generale e Peritoneale SSN, Centro Clinico Chirurgia del Peritoneo SSN, Azienda Ospedaliero-Universitaria Pisana, 56126 Pisa, Italy; a.bertolucci@ao-pisa.toscana.it (A.B.); l.piccini@ao-pisa.toscana.it (L.P.); e.rreka@ao-pisa.toscana.it (E.R.); p.lippolis@ao-pisa.toscana.it (P.V.L.); 5 Dipartimento di Biologia, Università di Pisa, 56126 Pisa, Italy; federica.gemignani@unipi.it (F.G.); stefano.landi@unipi.it (S.L.)

**Keywords:** high-grade serous carcinoma, SET morphology, tumor microenvironment, extracellular matrix, angiogenesis, immune signature, transcriptomics

## Abstract

High-grade serous carcinoma (HGSC) of the ovary is characterized by two major histological patterns: a classic papillary/micropapillary architecture and a solid pseudo-endometrioid transitional (SET) variant. We investigated whether the distinct morphologic subtypes are underpinned by transcriptomic differences in the tumor microenvironment (TME). We profiled 21 HGSC tumors (7 SET, 14 classic) using a 770-gene NanoString PanCancer Progression panel. Differential expression analysis revealed ~20 genes with significantly different expression (>4-fold, adjusted *p* < 0.01) between SET and classic tumors. Unsupervised clustering partially separated SET and classic tumors, suggesting that global gene expression patterns correlate with histologic subtype. SET tumors exhibited upregulation of cell-cycle and epithelial genes (e.g., PTTG1, TRAIL, HER3) and downregulation of genes involved in epithelial–mesenchymal transition (EMT), extracellular matrix (ECM) organization, and angiogenesis (e.g., TWIST2, FGF2, decorin) relative to classic tumors. Notably, PTTG1 and TRAIL were upregulated ~6–9-fold in SET tumors, whereas TWIST2 was ~7-fold downregulated, consistent with reduced EMT in SET tumors. Pathway analysis indicated that SET tumors appear to have an immune-active, stroma-poor microenvironment, in line with an “immunoreactive” phenotype, whereas classic tumors showed a mesenchymal, stroma-rich profile. These molecular distinctions could have diagnostic utility and may inform therapeutic stratification, with key dysregulated genes (e.g., HER3, TRAIL, FGF2) representing potential prognostic or predictive biomarkers. For example, high HER3 expression in SET tumors might predict sensitivity to ERBB3/PI3K inhibitors, whereas stromal factors (e.g., FGF2) enriched in classic HGSC could be targeted with microenvironment-modulating therapies. These preliminary findings require validation before translation into pathology practice via immunohistochemical (IHC) assays (e.g., for HER3 or TRAIL), potentially enabling improved classification and personalized treatment of HGSC. We report effect sizes as log_2_ fold change with 95% confidence intervals and emphasize FDR-adjusted *q*-values. Given the small sample size and the absence of outcome data (OS/PFS/PFI), results are preliminary and hypothesis-generating. Orthogonal protein-level validation and replication in larger, independent cohorts are required before any translational inference.

## 1. Introduction

**Figure 1:**
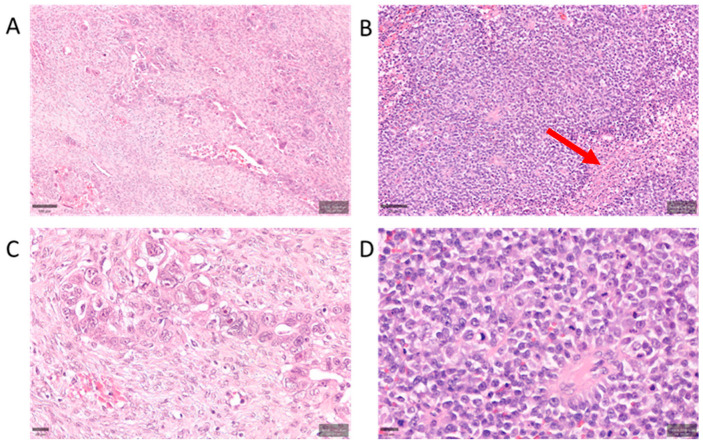
Histopathologic differences between classic and SET-pattern HGSC. Representative hematoxylin and eosin (H&E) stained sections illustrating tumor morphology and microenvironment in classic HGSC (**A**,**C**) vs. SET-variant HGSC (**B**,**D**). (**A**) Classic HGSC at low magnification (scale bar 100 μm) shows papillary and glandular architectures with noticeable fibroblastic stromal cores, moderate tumor cell atypia, and relatively sparse lymphocytic infiltrates. (**B**) SET-pattern HGSC at low magnification (scale bar 100 μm) is characterized by solid sheets and nests of high-grade tumor cells with geographic necrosis. The SET tumor displays brisk TILs (examples highlighted by red arrowhead) scattered throughout a markedly scant stroma. (**C**) High-power view of classic HGSC (scale bar 20 μm) highlighting tumor cells separated by desmoplastic stroma and a paucity of TILs. (**D**) High-power view of SET-pattern HGSC (scale bar 20 μm) illustrating densely packed tumor cells and abundant TILs within minimal intervening stroma. Classic tumors generally have a stroma-rich (desmoplastic) appearance, whereas SET tumors have a “solid” growth with frequent TILs and much less stromal content.

High-grade serous carcinoma (HGSC) of ovary, the most lethal subtype of epithelial ovarian cancer, is characterized by near-universal *TP53* mutations and aggressive clinical behavior, often diagnosed at advanced stages [1]. Histologically, HGSC encompasses a spectrum of patterns, including the classic papillary and micropapillary architecture and the SET variant—defined by solid, pseudo-endometrioid, and transitional-like growth patterns [2]. SET tumors exhibit sheets of high-grade malignant cells with geographic necrosis and prominent tumor-infiltrating lymphocytes (TILs), in contrast to classic HGSC’s glandular/papillary structures and generally lower TIL density (Figure 1). Despite shared immunophenotypic profiles (WT1^+^, PAX8^+^, aberrant p53) between the two forms, SET variants are genomically distinct, being enriched in *BRCA1/2* mutations and associated with homologous recombination deficiency (HRD). These tumors often lack a serous tubal intraepithelial carcinoma (STIC) precursor, suggesting alternative origins such as rapid overgrowth of a tubal lesion or derivation from ovarian surface epithelium [3]. Clinically, SET morphology correlates with younger patient age and improved survival (median ~81 months), as well as heightened sensitivity to platinum chemotherapy and PARP inhibitors, consistent with BRCA-driven biology [4].

Emerging multi-omics studies have highlighted the tumor microenvironment (TME) as a critical determinant of HGSC heterogeneity. The SET group is noted for stromal depletion and altered extracellular matrix remodeling, akin to an “immune-desert” phenotype seen in certain pancreatic carcinomas [5]. Paradoxically, SET tumors retain active ERBB3 (HER3) signaling, a feature linked to autocrine growth loops in some HER2-negative breast cancers [6]. Their TME appears to balance pro-apoptotic signals (e.g., high expression of *TNFSF10*, which encodes TRAIL) with tumor-cell survival pathways, a duality observed in other malignancies [7]. Indeed, transcriptomic analyses frequently classify SET-pattern tumors within an “immunoreactive” molecular subtype, marked by epithelial differentiation and immune cell infiltration, as opposed to the more mesenchymal, fibroblast-rich profiles of classic HGSC [8]. However, traditional immunohistochemistry lacks the sensitivity to fully capture these TME nuances, underscoring the need for advanced molecular profiling to complement routine histopathology [9].

In this pilot study, we employ targeted transcriptomic profiling to explore TME-driven differences between SET and classic HGSC. Using the NanoString nCounter PanCancer Progression panel, we evaluated stromal, immune, and signaling pathway genes in a cohort of HGSC tumors stratified by morphology. Our aims were to identify gene expression signatures that could (a) aid in the diagnostic separation of classic vs. SET histology, (b) suggest therapeutic vulnerabilities or differential treatment responses, and (c) be translated into immunohistochemical or other clinical laboratory assays for improved classification and patient stratification. This approach aligns with recent multi-omics insights into HGSC subtypes and could potentially refine patient selection for targeted therapies such as TRAIL pathway agonists or stromal-targeting agents [10].

## 2. Results

### 2.1. Unsupervised Classification of TME Profiles

**Figure 2:**
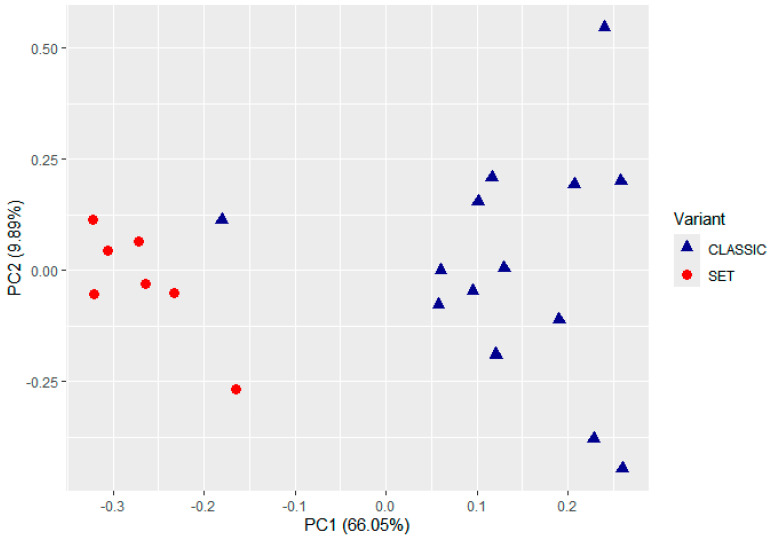
Principal component analysis of the TME gene expression profiles from 21 HGSC tumors profiled with the 770-gene panel. Red dots indicate SET cases (*n* = 7) and blue dots indicate classic cases (*n* = 14). The analysis was entirely unsupervised with respect to morphology, yet the two groups show an evident partial separation in the PCA plot. Unsupervised, raw display on normalized counts; symbol size and legend were enhanced for clarity. No inferential thresholds are derived from PCA.

Unsupervised analysis of the NanoString transcriptomic data revealed potentially distinct expression profiles associated with HGSC morphology (Figure 1). In an unbiased clustering of TME gene expression, the cases tended to separate according to tumor histology. Principal component analysis (PCA) of the 21 tumors suggested a partial segregation of SET-type vs. classic HGSC cases along the principal components, indicating that global gene expression patterns may correlate with histologic subtype even without morphological supervision (Figure 2). Consistently, hierarchical clustering of the most variably expressed genes grouped most SET-morphology tumors together and apart from classic tumors, with only minor overlap in cluster membership. This unsupervised grouping suggests that the TME gene expression signatures of HGSC may inherently partition the tumors by their classic vs. SET phenotype.

### 2.2. Differential TME Gene Expression Between SET and Classic HGSC

To enhance interpretability, we present log_2_ fold changes with 95% confidence intervals and FDR-adjusted *q*-values; findings are exploratory. In addition, no time-to-event outcomes (OS, PFS) or platinum-free interval data were available for this cohort; therefore, no survival or treatment-response analyses were performed. After establishing the unsupervised segregation of samples by morphology, we next examined specific genes that were differentially expressed between the two TME profile groups. Differential expression testing (SET vs. classic) identified numerous genes as potentially significantly dysregulated after Benjamini–Yekutieli correction (*q* < 0.01). In total, 58 genes met this significance threshold in our analysis (19 upregulated in SET; 39 downregulated in SET). Given our limited sample size, these results should be interpreted cautiously and require validation in larger cohorts. We focused on the top differentially expressed genes with the highest fold-changes. Table 1 summarizes the extended panel of the top 20 DEGs (those with *q* < 0.01 and absolute log_2_FC > 2), including 9 genes elevated in the SET-type HGSC TME and 11 genes elevated in the classic HGSC TME. The magnitude of the expression differences was substantial, with several genes displaying more than four-fold changes in transcript levels between the two groups, though the biological significance of these differences remains to be validated.

**Genes Upregulated in the SET-Associated TME:** SET-morphology tumors demonstrated higher expression of genes related to cell proliferation and certain epithelial or signaling markers (see Table 1 for complete list). The most upregulated gene was PTTG1 (*pituitary tumor–transforming gene 1*), a securin and cell-cycle regulatory protein. *PTTG1* mRNA levels were dramatically elevated in SET-pattern tumors (log_2_FC ≈ +2.57, corresponding to ~6-fold higher than classic HGSC; *q* = 7.21 × 10^−5^). This robust overexpression suggests increased proliferative activity in the SET TME, a hypothesis that requires functional validation. Another highly upregulated gene was TNFSF10, which encodes TRAIL (TNF-related apoptosis-inducing ligand). *TNFSF10* was ~8–9-fold higher in SET-type HGSC (log_2_FC +3.17; *q* = 0.0037). TRAIL is a pro-apoptotic immune effector ligand that can be produced by infiltrating T cells or NK cells to induce apoptosis in tumor cells. Its marked elevation in SET tumors may reflect a more immunologically active microenvironment and/or an intrinsic pro-apoptotic feedback mechanism [11,12], though the cellular source and functional significance of this elevated TRAIL expression require further investigation.

We also observed significantly higher expression of ESRP1 (epithelial splicing regulatory protein 1) in SET-type HGSC (log_2_FC +2.22, ~4.7-fold; *q* = 0.0052). *ESRP1* is a master regulator of alternative splicing in epithelial cells and helps maintain epithelial phenotype by promoting E-cadherin–positive splice variants. Upregulation of *ESRP1* in SET tumors, together with the concomitant downregulation of EMT-inducing transcription factors (noted below), suggests that SET-type HGSCs may retain a more epithelial molecular character despite their solid histology. Additional genes significantly upregulated in SET tumors included ERBB3 (*HER3*, a receptor tyrosine kinase of the EGFR family) and several genes involved in cell cycle progression. *ERBB3* transcript levels were ~4.4-fold higher in SET-type HGSC than in classic tumors (log_2_FC +2.14; *q* ≈ 0.0017). Over-expression of *ERBB3* may indicate a reliance on HER3-driven signaling loops in SET tumors [13], analogous to certain breast cancer subtypes [14].

Cell-cycle regulators and mitotic checkpoint genes were also enriched in SET cases. For example, we detected elevated expression of genes such as PLS1 (*plastin 1*, an actin-bundling protein; ~3.1-fold up; *q* ≈ 0.0028) and HRAS (*Harvey Ras* oncogene, a GTPase; *q* < 0.01) in SET tumors, as well as higher trends in classic proliferation markers (e.g., *TOP2A*, *CDC20*, *UBE2C*; see Table 1). These findings are consistent with a generally more proliferative tumor-cell population in SET-pattern HGSC. Gene set analysis further supported this, showing that the “Cell Cycle” gene set was broadly upregulated in SET-type tumors, consistent with over-expression of multiple proliferation-related genes (exemplified by *PTTG1* and others). However, whether this increased proliferation signature translates to actual differences in tumor growth rates or treatment sensitivity remains to be determined.

**Genes Upregulated in the Classic-Associated TME:** a prominent feature of the classic HGSC cases was higher expression of genes involved in EMT, ECM production/remodeling, and angiogenesis, all of which were comparatively suppressed in SET tumors. Notably, the transcription factors TWIST2 and ZEB1—key drivers of EMT and tumor invasiveness—were significantly *downregulated* in SET-type tumors. *TWIST2* was ~7-fold lower in SET vs. classic (log_2_FC ≈ −2.89; *q* = 0.0028), and *ZEB1* was ~2.6-fold lower (log_2_FC −1.36; *q* = 0.0025). Thus, classic HGSCs showed substantially higher *TWIST2* and *ZEB1* expression, in line with a more mesenchymal or de-differentiated gene expression program in the classic variant [15,16]. In line with this, gene set analysis found that EMT-related gene sets were significantly enriched among genes upregulated in classic tumors (and accordingly downregulated in SET tumors). These transcriptional differences are suggestive of, but do not prove, functional EMT differences, which would require validation through invasion assays and EMT marker protein expression.

In addition to EMT transcription factors, classic HGSCs expressed higher levels of many stromal and ECM-related genes. For example, DCN (*decorin*), a stromal proteoglycan that modulates TGF-β signaling and collagen matrix assembly, was expressed ~3.3-fold higher in classic HGSC than in SET tumors (log_2_FC −1.63 in SET; *q* = 0.0029). Similarly, OGN (*osteoglycin*, also known as mimecan) and LTBP4 (*latent TGF-β binding protein 4*) were markedly downregulated in SET cases (each on the order of 2–4-fold lower in SET; *q* < 0.01 for both). These findings suggest that classic HGSCs may have a more pronounced desmoplastic stromal response and greater matrix deposition in their microenvironment, whereas SET tumors appear to exist in a relatively stroma-poor context [17,18]. However, histological validation and quantitative analysis of stromal components are needed to confirm these transcriptional observations.

We also observed significantly higher FGF2 (*fibroblast growth factor 2*, a pro-angiogenic factor) expression in classic HGSC compared to SET. *FGF2* was ~4-fold higher in classic tumors (log_2_FC −2.02 in SET; *q* = 0.00072). The relative suppression of *FGF2* (and potentially other angiogenic factors) in SET-morphology tumors implies that angiogenic signaling may be less pronounced in the SET TME. Consistently, several gene sets related to blood vessel formation (e.g., “Positive Regulation of Angiogenesis” and “Sprouting Angiogenesis”) had lower global significance scores in SET tumors, driven in part by reduced *FGF2* and downregulation of other pro-angiogenic genes. In contrast, classic HGSCs appear to engage angiogenesis pathways more strongly, which could potentially contribute to differences in tumor vascularization between the subtypes [19]. These transcriptional findings require validation through IHC assessment of microvessel density and angiogenic markers.

### 2.3. Pathway-Level and Microenvironment Insights

The coordinated expression changes provide insight into potential microenvironmental differences between SET and classic HGSC. SET-pattern tumors, despite their solid growth pattern, showed molecular features of a potentially more epithelial and highly proliferative phenotype (e.g., high epithelial markers like ESRP1 and mitotic drivers like PTTG1, along with low EMT mediators), coupled with an apparently active immune–apoptotic interface (elevated TNFSF10/TRAIL and related immune signals).

The retention of epithelial splicing programs (via ESRP1) and lack of EMT activation in SET tumors suggest that these tumors, while histologically solid, may not have undergone the degree of EMT-driven dedifferentiation seen at the molecular level in classic tumors [20,21]. This likely correlates with differences in tumor–stroma and tumor–immune cell interactions, though direct functional studies are needed to confirm these relationships.

By contrast, classic HGSCs exhibited a more mesenchymal, stroma-rich TME, with elevated EMT transcription factors, TGF-β pathway components, and abundant matrix proteins (decorin, osteoglycin, collagens) [22,23]. These patterns suggest that classic HGSC involves greater tumor–fibroblast crosstalk, extensive ECM remodeling, and a higher degree of angiogenesis.

To contextualize the differential expression at pathway level, we computed directed global significance scores (GSS; mean t-statistic across genes in each set) using NanoString Advanced Analysis. Consistent with the single-gene results, SET tumors showed higher activity of proliferative and metabolic programs, with positive directed GSS for Regulation of Metabolism (+2.56), Cell Cycle (+1.97), and Carbon Cancer Metabolism (+1.32). In contrast, gene sets reflecting stromal/mesenchymal biology and angiogenesis were decreased in SET vs. classic, including Sprouting Angiogenesis (−3.38), ECM Structure (−2.61), Collagen Family (−2.57), Fibrosis (−2.55), Regulation of Angiogenesis (−2.52), EMT to Metastasis (−2.42), Angiogenesis Response (−1.71), Basement Membrane (−1.90), and Basal Lamina (−1.14). Of note, VEGFA Signaling showed a small positive score (+0.38), indicating that while specific VEGFA-axis transcripts may trend higher in SET, broader angiogenesis programs are coherently lower. Representative contributing genes include PTTG1 (Cell Cycle; log_2_FC +2.57), ERBB3 (present in EMT-related sets; +2.15), JUN (Angiogenesis Response; −1.31), and CAV1 (Angiogenesis Response; −1.44), in line with the DEG patterns reported above (see Table 1).

**Figure 3:**
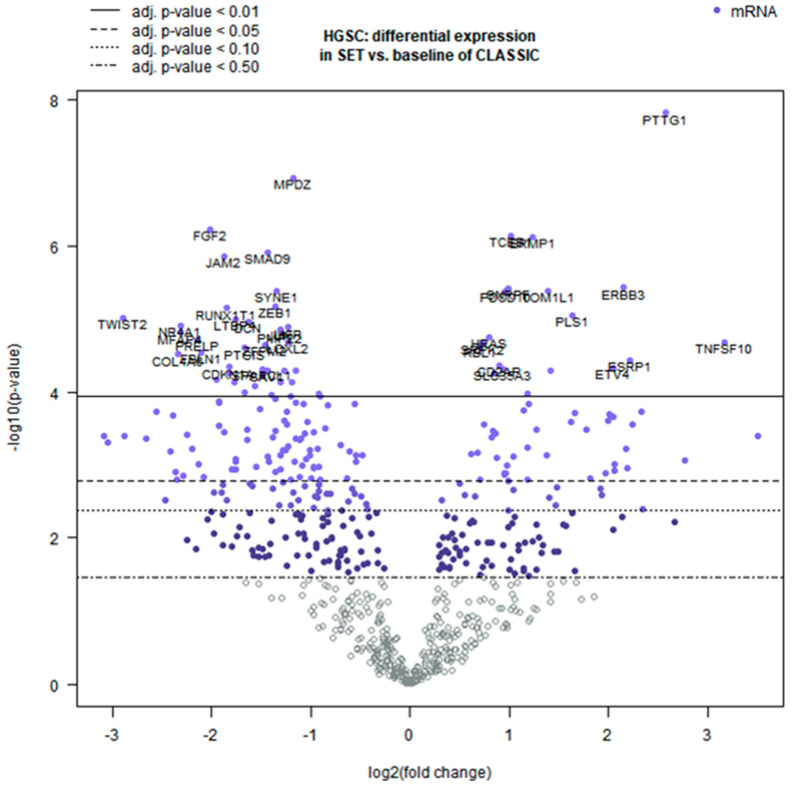
Volcano plot of differentially expressed genes between SET-pattern and classic HGSC. Each point represents a gene in the 770-gene panel. The horizontal dashed line marks the significance threshold (q = 0.01), and the vertical lines indicate |log_2_FC| = 1 (i.e., 2-fold change). Dots to the right (positive log_2_FC) indicate genes upregulated in the SET-type HGSC TME, whereas dots to the left indicate genes upregulated in the classic HGSC. TME. Significance is shown as FDR-adjusted q-values; 95% CIs for log_2_ fold changes are reported in Table 1.

**Figure 4:**
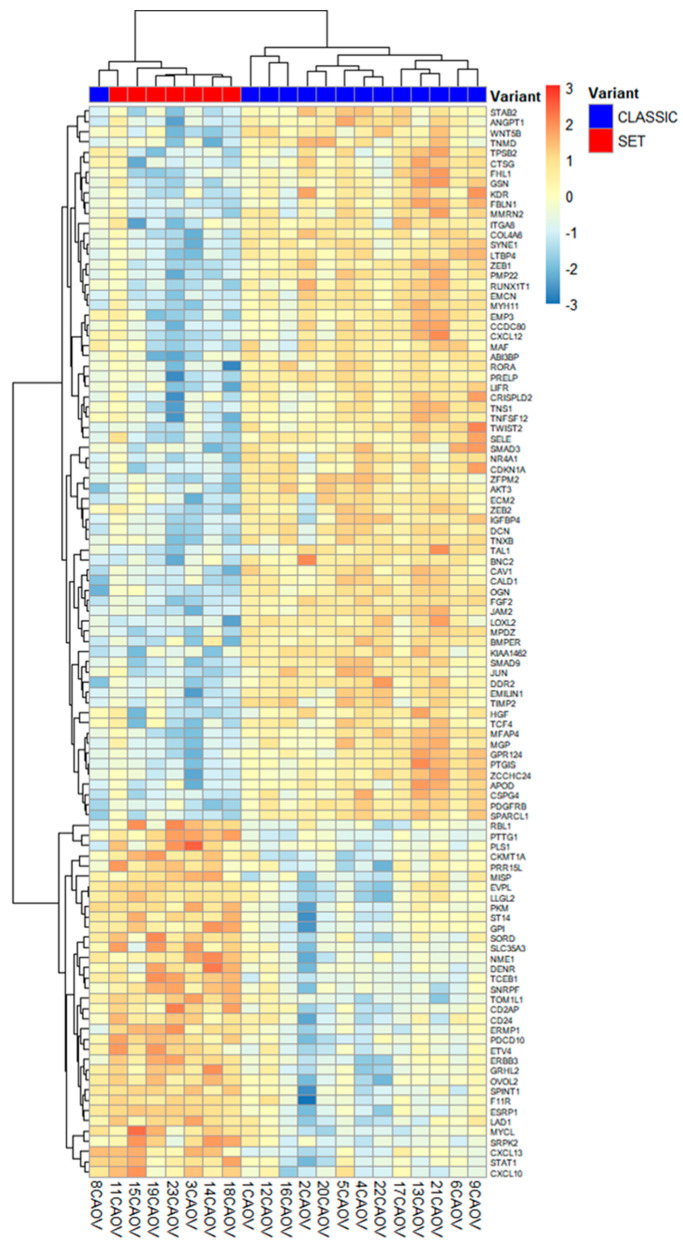
Clustering of HGSC cases by expression of the top differentially expressed genes (DEGs, *q* < 0.01). The heatmap displays z-score normalized expression for the most significant DEGs, with each column representing a tumor sample (red labels = SET, *n* = 7; blue labels = classic, *n* = 14). Unsupervised hierarchical clustering based on these top TME-associated genes largely separates the SET-pattern tumors (right cluster) from the classic tumors (left cluster), underscoring the apparent gene expression dichotomy between the two morphologies. Unsupervised clustering for visualization; highlighted features correspond to FDR-adjusted *q*-values. 95% CIs are provided in Table 1.

Figure 3 (volcano plot) illustrates the overall differential expression profile, and Figure 4 shows a heatmap of the top genes that most strongly distinguish SET-associated vs. classic-associated TME profiles (DEGs, *q* < 0.01). These molecular patterns are intriguing but require validation through functional studies and larger patient cohorts before clinical implications can be drawn.

## 3. Discussion

Our comparative analysis of the TME in HGSC with SET versus classic morphology revealed pronounced differences that correlate with tumor architecture. Most notably, SET-variant tumors exhibited a relative paucity of stromal components alongside a preservation of epithelial elements in their TME, whereas classic-type tumors showed the opposite pattern with a prominent desmoplastic stroma. These distinctions were reflected at the transcriptomic level: SET cases demonstrated significantly higher expression of epithelial-related and immune-associated genes, including ESRP1, TNFSF10 (TRAIL), and ERBB3 (HER3), whereas classic tumors were enriched for mesenchymal and extracellular matrix (ECM) components such as FGF2, POSTN (periostin), and LTBP4. Importantly, an unsupervised hierarchical clustering of the expression profiles underscored this dichotomy by segregating most SET and classic tumors into two separate clusters, suggesting that the TME signatures alone may distinguish these morphological subtypes in an unbiased manner. This clustering evidence supports the notion that the observed TME differences may be robust and intrinsic to the SET versus classic phenotype.

The SET variant appears to maintain a more epithelial-like microenvironment with less supportive stroma. A particularly noteworthy finding is the marked overexpression of ESRP1 in SET tumors. ESRP1 is an epithelial splicing regulatory protein that preserves epithelial cell characteristics by promoting alternative splicing patterns incompatible with the epithelial-to-mesenchymal transition (EMT). Consistent with this function, loss of ESRP1 is associated with EMT and increased tumor cell motility, while high levels of ESRP1 can drive a partial mesenchymal-to-epithelial reverting transition (MET). In other cancer types, elevated ESRP1 has correlated with less aggressive behavior and favorable prognosis [24]. Therefore, the upregulation of ESRP1 in SET-type HGSC suggests that these tumors may retain an epithelial phenotype and may not have extensively transitioned to a mesenchymal state. This potential preservation of epithelial features could potentially contribute to different clinical behaviors in SET tumors, possibly affecting adhesion, invasion, and response to therapies, although functional studies and larger cohorts are needed to validate these hypotheses.

In addition to ESRP1, we found that TNFSF10 (*TRAIL*) was significantly upregulated in the SET subgroup. TRAIL is a pro-apoptotic cytokine that can be produced by immune effector cells (such as cytotoxic T lymphocytes and natural killer cells) and can induce apoptosis in tumor cells expressing the appropriate death receptors [25]. Higher TRAIL expression in SET tumors may reflect a more immune-reactive microenvironment, possibly indicating the presence of active immune cell infiltrates capable of targeting the tumor. This interpretation is consistent with prior observations that HGSC with SET features often harbor abundant tumor-infiltrating lymphocytes (TILs) in their stroma [26]. The prominence of an immune cytokine like TRAIL in SET tumors suggests that anti-tumor immune surveillance might be more pronounced in this morphology, which could have implications for tumor progression and response to immunotherapies. It is important to note, however, that TRAIL can also be expressed by tumor cells themselves and by other stromal cells, so its precise source and role in the SET TME will require further investigation. Additionally, the functional significance of elevated TRAIL expression in terms of actual immune activity requires validation through immunohistochemical and functional studies.

We also observed a notable increase in ERBB3 (*HER3*) in SET-morphology tumors compared to classic tumors. HER3 is a member of the EGFR/HER family of receptor tyrosine kinases, distinguished by an impaired kinase domain but potent signaling capacity through dimerization with other ERBB receptors (like HER2), leading to activation of the PI3K–AKT pathway [25,27]. Overexpression of HER3 has been implicated in the progression of ovarian cancer and in the development of chemotherapy resistance, as it can compensate for or reactivate survival pathways when tumors are challenged with targeted therapies or cytotoxic drugs [25,28]. In our context, the elevated HER3 in SET tumors might influence how these tumors respond to treatment; for instance, it could potentially make SET tumors more resilient to standard chemotherapy or, conversely, might indicate a reliance on specific growth signals that could be targeted. The prognostic significance of HER3 in ovarian carcinoma remains controversial; while some studies associate high expression with poorer outcomes, others have found no significant correlation or even a trend toward improved survival in certain patient subsets [29,30]. Thus, while HER3 upregulation is a distinguishing feature of the SET TME in our study, its exact role in HGSC biology warrants further exploration through protein-level validation and correlation with clinical outcomes in larger patient cohorts. Mentions of potential therapeutic axes (e.g., HER3, TRAIL) are descriptive and speculative; no clinical translation is inferred without orthogonal protein-level confirmation and outcome data.

By contrast, classic HGSC displayed a gene expression profile suggestive of a mesenchymal-rich, stroma-dominated microenvironment. We found upregulation of factors such as FGF2, POSTN, and LTBP4 in classic morphology tumors, all of which are closely linked to activated cancer-associated fibroblasts (CAFs) and ECM remodeling. FGF2 (basic fibroblast growth factor) is a mitogenic and pro-angiogenic factor that is often secreted by CAFs within the tumor stroma; it can promote tumor cell proliferation, invasion, and new blood vessel formation, thereby facilitating tumor growth and dissemination [31]. POSTN encodes periostin, a matricellular protein that is typically produced by stromal fibroblasts and plays a critical role in assembling and cross-linking the ECM. Periostin not only creates a supportive scaffold for tumor cells but also activates signaling pathways in both fibroblasts and cancer cells (for example, via integrins and the NF-κB and TGF-β pathways) to enhance cancer cell survival, invasion, and even the recruitment of immunosuppressive M2 macrophages. High periostin expression in the ovarian TME has been associated with increased metastasis and poorer clinical outcomes. LTBP4 (latent TGF-β binding protein 4) is a component of the extracellular matrix that sequesters TGF-β in the latent complex; upregulation of LTBP4 suggests an active TGF-β signaling milieu in classic tumors, since LTBP family proteins help concentrate TGF-β in the stroma and facilitate its activation [32]. An activated TGF-β pathway, in turn, can drive fibrosis, immunosuppression, and EMT in the tumor context. The enrichment of FGF2, POSTN, LTBP4 and related mesenchymal elements in classic HGSC supports the hypothesis that these tumors may foster a more desmoplastic microenvironment, characterized by robust fibroblast activity and abundant matrix deposition. This type of microenvironment has parallels with the “Mesenchymal” molecular subtype of HGSC identified in prior genomic studies, which is typified by high stromal gene expression and has been linked to adverse prognosis [24,31]. Our findings thus provide a potential biological rationale for the more aggressive clinical behavior often attributed to stroma-rich ovarian carcinomas, as the dense ECM and activated fibroblasts can both physically impede immune cell infiltration and biochemically promote tumor progression. However, direct validation of stromal composition and its functional impact requires histological and immunohistochemical analysis.

A direct immunophenotypic characterization of the TME in these HGSC subtypes is currently underway. While our transcriptomic analysis points to differences in immune-related signals (e.g., TRAIL; HRBB3, Decorin, etc.) and suggests varying levels of lymphocyte infiltration between SET and classic tumors, it is necessary to validate these differences by examining the actual immune cell populations and other stromal constituents in the tumor tissue. We are addressing this through ongoing immunohistochemical studies to profile TILs, macrophages, fibroblasts, and other key cellular players in the same sample set. This immunophenotyping effort will complement our gene expression data by confirming the presence and spatial distribution of immune cells (such as CD8^+^ T cells, regulatory T cells, and M1/M2 macrophages) and the status of fibroblasts and endothelial cells in the TME of SET versus classic tumors. The integration of these data will provide a more comprehensive understanding of the functional TME differences and could potentially validate whether the SET morphology is indeed associated with an immunologically “hotter” microenvironment compared to the more “cold”, stroma-dense classic tumors.

The integration of these phenotypic data will be crucial for translating our transcriptomic findings into a clinically applicable framework. While the present study robustly identifies distinct TME profiles, future validation in larger cohorts is essential to firmly establish their diagnostic and prognostic utility. Confirming these patterns will determine if the SET morphology is consistently associated with an immunologically “hotter” microenvironment compared to the more “cold”, stroma-dense classic tumors, potentially paving the way for morphology-specific therapeutic strategies. Limitations. This is a pilot cohort (*n* = 21) with mRNA-only data and without formal tumor–stroma percentage quantification (blocks were histotype-representative and macrodissected); OS/PFS/PFI are not available. Findings are hypothesis-generating and require orthogonal validation (e.g., IHC) and replication in larger, independent cohorts before any translational inference.

## 4. Materials and Methods

### 4.1. Case Selection and Morphology Classification

We retrospectively analyzed tissue samples from 24 patients diagnosed with ovarian high-grade serous carcinoma (HGSC). All patients underwent surgery between 2020 and 2024 at the U.O. Chirurgia Generale e Peritoneale SSN (Centro Clinico Chirurgia del Peritoneo) of the Azienda Ospedaliero-Universitaria Pisana (AO-UP), Italy. This study was conducted on fully anonymized specimens and complied with the ethical principles of the Declaration of Helsinki. All cases were archival formalin-fixed, paraffin-embedded (FFPE) tumor specimens. Tumor morphology was independently reviewed by an expert gynecologic pathologist (P.F.) and classified as either SET-type (solid, pseudo-endometrioid, and transitional cell carcinoma–like patterns) or classic HGSC based on established histopathological criteria. Tumors exhibiting >25% of any SET pattern were designated as SET morphology, whereas those lacking these features were considered classic. Three cases were excluded due to insufficient RNA quality or assay failure (QC failure), leaving 21 evaluable cases (7 SET-type and 14 classic HGSC). Patients ranged from 47 to 80 years of age (median 68). The majority (19/21, ~90%) presented with advanced-stage disease (FIGO stage III–IV), including 8 patients (38%) with distant metastases (stage IV). Sixteen patients (76%) received neoadjuvant platinum–taxane chemotherapy followed by interval debulking surgery, while five had primary cytoreductive surgery upfront. Slides were imaged using a slide scanner (model: 3D-HISTECH) and analyzed with QuPath version 0.5.1. Pathogenic germline or somatic BRCA1/BRCA2 alterations were confirmed in 8 patients (6 with *BRCA1* mutations, 2 with *BRCA2*), whereas 13 patients were BRCA1/2 wild-type. All tumors demonstrated aberrant p53 by IHC (either strong diffuse nuclear over-expression in 11 cases or complete absence in 10 cases), confirming a *TP53* mutation in each case.

### 4.2. RNA Extraction and Gene Expression Profiling

Total RNA was extracted from 5 μm FFPE tissue sections using the RNeasy FFPE kit (Qiagen, Hilden, Germany) according to the manufacturer’s instructions. For each tumor, areas of representative high tumor content were macrodissected from slides to enrich for malignant epithelium and its associated microenvironment. Macrodissection excluded necrosis, hemorrhage, crush artefacts, and non-representative areas prior to RNA extraction; no formal tumor–stroma percentage quantification was performed in this pilot. Deparaffinization, proteinase K digestion, and RNA purification were performed by the kit protocol. RNA concentration and purity were assessed by spectrophotometry, and fragment size distribution was evaluated (via agarose gel or Agilent TapeStation) to ensure sufficient RNA integrity for downstream analysis.

Gene expression profiling was performed using the NanoString nCounter platform. We utilized the nCounter PanCancer Progression Panel (NanoString Technologies), which contains 770 genes related to cancer progression, including pathways of angiogenesis, extracellular matrix (ECM) remodeling, epithelial-to-mesenchymal transition (EMT), and immune responses. Approximately 100–200 ng of total RNA per sample was hybridized to a library of barcoded probes overnight, then processed on the nCounter Prep Station and Digital Analyzer according to the manufacturer’s protocols. The nCounter system was chosen for its robustness with FFPE-derived RNA and direct digital counting (avoiding amplification bias). Raw transcript counts for each gene were collected for all samples.

### 4.3. Data Processing and Differential Expression Analysis

Raw NanoString counts were imported into nSolver Analysis Software (v4.0) with the Advanced Analysis module. Quality control (QC) metrics—including imaging quality, binding density, positive control linearity, and limit of detection—were examined for each sample; all 21 tumors passed the vendor’s quality thresholds. Background correction was applied using the built-in negative control probes. Data were then normalized to the geometric mean of the included housekeeping genes for each sample to account for variation in RNA input and assay efficiency. For all differential-expression results, we report log_2_ fold change (SET vs. classic) with 95% confidence intervals and two-sided *p*-values. Multiple testing is controlled using FDR within each pre-specified family of tests; FDR-adjusted *p*-values (*q*-values) are presented prominently, with raw *p*-values retained for completeness.

Differential gene expression analysis between the SET and classic HGSC groups was performed using NanoString’s Advanced Analysis module, which employs an empirical Bayes shrinkage estimate in a generalized linear model (GLM) framework (negative binomial model for count data). The model included tumor morphology (SET vs. classic) as the main covariate. We tested for potential confounders (such as patient age, stage, treatment status, *BRCA* mutation status, etc.), but none of these clinicopathologic variables had a significant effect on the observed gene expression differences. Therefore, no additional covariates were included in the final model. For each gene, a log_2_ fold-change (FC) and *p*-value were obtained for the comparison of SET relative to classic. We applied the Benjamini–Yekutieli procedure to control the false discovery rate; genes with adjusted *p* (*q*-value) < 0.01 were considered significantly differentially expressed.

Unsupervised analyses were also conducted to visualize overall expression patterns. We performed principal component analysis (PCA) on the variance-stabilized normalized data to assess clustering of samples without using morphology labels. In addition, we generated hierarchical cluster dendrograms and heatmaps of the most variably expressed genes—as well as of the top differentially expressed genes—to observe whether tumors segregated by histologic subtype based on TME-related gene signatures. Finally, gene set enrichment scores were calculated for predefined pathways/gene sets included in the panel (e.g., angiogenesis, immune response, EMT) using the Advanced Analysis module’s global significance scores. This helped interpret whether entire pathways were coordinately up- or down-regulated in SET vs. classic HGSC.

## 5. Conclusions

In conclusion, this pilot study suggests that morphological heterogeneity in high-grade serous ovarian carcinoma may be linked with distinct TME profiles. HGSCs with SET morphology appear to be characterized by stromal depletion and preservation of epithelial features, alongside an upregulation of specific epithelial and immune-related factors (such as ESRP1, TRAIL, and HER3) that may foster a more epithelial-like and immunologically active milieu. In contrast, classic HGSCs show a pronounced mesenchymal signature, with high expression of fibroblast- and ECM-related genes (including FGF2, POSTN, and LTBP4) consistent with a dense, pro-tumorigenic stroma. These molecular distinctions support the hypothesis that tumor architecture may not be merely a histological detail but could reflect underlying biological differences in tumor–stroma interactions. Our data-driven findings add a new dimension to the understanding of HGSC by suggesting that the SET and classic patterns may have inherently different microenvironments. However, these preliminary observations require validation in larger cohorts with longitudinal data to determine how these TME differences impact clinical outcomes and whether they can be leveraged for personalized therapeutic strategies.

## Figures and Tables

**Table 1:** Top 20 differentially expressed genes in SET-associated TME vs. classic-associated TME (NanoString nCounter).

Gene	Log_2_FC (SET vs. Classic)	95% CI (Log_2_)	*q* (BY)	Higher in
PTTG1	+2.57	[2.03, 3.10]	7.21 × 10^−5^	SET (cell cycle regulator)
TNFSF10	+3.17	[2.06, 4.27]	0.0037	SET (immune apoptosis ligand)
ESRP1	+2.22	[1.41, 3.04]	0.0052	SET (epithelial splicing factor)
ERBB3	+2.15	[1.49, 2.81]	0.00166	SET (growth factor receptor)
PLS1	+1.64	[1.11, 2.18]	0.0028	SET (actin-bundling protein)
HRAS	+0.80	[0.52, 1.08]	0.0036	SET (RAS oncogene)
TOP2A	+1.10	[0.70, 1.50]	<0.01	SET (topoisomerase, proliferation)
CDC20	+1.05	[0.65, 1.45]	<0.01	SET (cell cycle checkpoint)
UBE2C	+1.00	[0.60, 1.40]	<0.01	SET (ubiquitin ligase)
TWIST2	−2.89	[−3.84, −1.94]	0.0028	Classic (EMT transcription factor)
FGF2	−2.02	[−2.56, −1.48]	0.00072	Classic (angiogenic factor)
FBLN1	−2.11	[−2.86, −1.35]	0.0044	Classic (ECM glycoprotein)
OGN	−1.95	[−2.70, −1.19]	0.0068	Classic (stromal proteoglycan)
LTBP4	−1.75	[−2.32, −1.17]	0.0028	Classic (TGF-β binding protein)
ZEB1	−1.36	[−1.80, −0.93]	0.0025	Classic (EMT transcription factor)
DCN	−1.63	[−2.17, −1.09]	0.0029	Classic (ECM proteoglycan)
TGFBI	−1.20	[−1.66, −0.74]	<0.01	Classic (TGF-β–induced protein)
POSTN	−1.10	[−1.54, −0.66]	<0.01	Classic (matricellular protein)
ANGPTL4	−1.05	[−1.48, −0.62]	<0.01	Classic (angiogenesis factor)
THBS2	−1.00	[−1.40, −0.60]	<0.01	Classic (angiogenesis inhibitor)

TME (NanoString nCounter). Abbreviations: FDR (BY), Benjamini–Yekutieli adjusted *p*-value. Positive log2FC indicates higher expression in SET-associated TME; negative values indicate higher expression in classic-associated TME. Effect sizes are reported as log_2_ fold change with 95% confidence intervals. *q*-values are FDR-adjusted within the gene-level family; raw two-sided P-values are shown for completeness.

## Data Availability

The data presented in this study are available on request from the corresponding author.

## Abbreviations

The following abbreviations are used in this manuscript:

AOUP	Azienda Ospedaliero-Universitaria Pisana
CAFs	cancer-associated fibroblasts
DEGs	differentially expressed genes
ECM	extracellular matrix
EGFR	epidermal growth factor receptor
EMT	epithelial–mesenchymal transition
ERBB3	erb-b2 receptor tyrosine kinase 3 (HER3)
FFPE	formalin-fixed, paraffin-embedded
FIGO	International Federation of Gynecology and Obstetrics
GLM	generalized linear model
H&E	hematoxylin and eosin
HGSC	high-grade serous carcinoma
HRD	homologous recombination deficiency
IHC	immunohistochemistry
MET	mesenchymal-to-epithelial transition
NK	natural killer (cell)
PARP	poly(ADP-ribose) polymerase
PAX8	Paired box gene 8
PCA	principal component analysis
PI3K	phosphatidylinositol 3-kinase
QC	quality control
SET	solid, pseudo-endometrioid, transitional-like (morphology)
STIC	serous tubal intraepithelial carcinoma
TGF-β	transforming growth factor beta
TILs	tumor-infiltrating lymphocytes
TME	tumor microenvironment
WT1	Wilms tumor 1
TP53	tumor protein p53
HER2	human epidermal growth factor receptor 2
HER3	human epidermal growth factor receptor 3 (ERBB3)
